# Community engagement and the importance of partnerships within the Great Lakes Areas of Concern program: A mixed-methods case study^☆^

**DOI:** 10.1016/j.jglr.2022.08.005

**Published:** 2022-12

**Authors:** Alison Rentschler, Kathleen C. Williams

**Affiliations:** a University of Michigan, 440 Church Street, Ann Arbor, MI 48109, USA; b United States Environmental Protection Agency Great Lakes Toxicology and Ecology Division, 6201 Congdon Blvd, Duluth, MN 55804, USA

**Keywords:** Areas of Concern program, Community engagement, Partnerships

## Abstract

The Great Lakes Areas of Concern (AOC) program was created through amendments to the Great Lakes Water Quality Agreement (GLWQA) in 1987 to restore contaminated sites using an ecosystem-based approach. This program represents one of the first instances of ecosystem-based management (EBM) in the Great Lakes region with a specific focus on the inclusion of the public and local stakeholders in the process. Despite official language incorporating EBM in the AOC program, implementation of these practices has not been consistent across AOCs given differences in local arrangements of Public Advisory Councils (PACs), approaches to community engagement, and environmental problems. To better understand community engagement in these complex AOCs, this research investigated community, PAC, and state agency perspectives in three AOCs in Michigan: the Kalamazoo River, Saginaw River and Bay, and Rouge River AOCs. We gathered data through interviews, focus groups, and participatory observations with community members, PAC members, and state officials in each AOC. Findings indicate that communities in these areas have minimal connection to the AOC program and PACs. Community members tended to have greater connection to local organizations that provide a variety of opportunities for community members to engage with their environment in ways they value. To better connect the public to the AOC program, PACs may benefit from intentional partnerships with community organizations to increase community engagement. To consistently bolster community engagement in AOCs, we further recommend that state agencies provide additional resources to improve connection to local communities.

## Introduction

The Great Lakes Areas of Concern (AOC) program was created to restore degraded ecosystems through the removal of toxic substances from impaired water resources utilizing an ecosystem-based approach (Sproule-Jones, 2002). Communities may revitalize after pollutant remediation and ecosystem restoration occur. Formalized through the US-Canada Great Lakes Water Quality Agreement (GLWQA) amendments of 1987, the AOC program represents the first formal effort to implement an ecosystem approach within the Great Lakes region and introduces stakeholder involvement as integral to progress (MacKenzie, 1993; Sproule-Jones, 2002). The 1987 amendments to the GLWQA outline direct stakeholder involvement as essential to environmental planning and remediation: “The Parties, in cooperation with the State and Provincial Governments, shall ensure that the public is consulted in the development and adoption of the Specific Objectives,” (IJC, 1988).

**Fig. 1.**
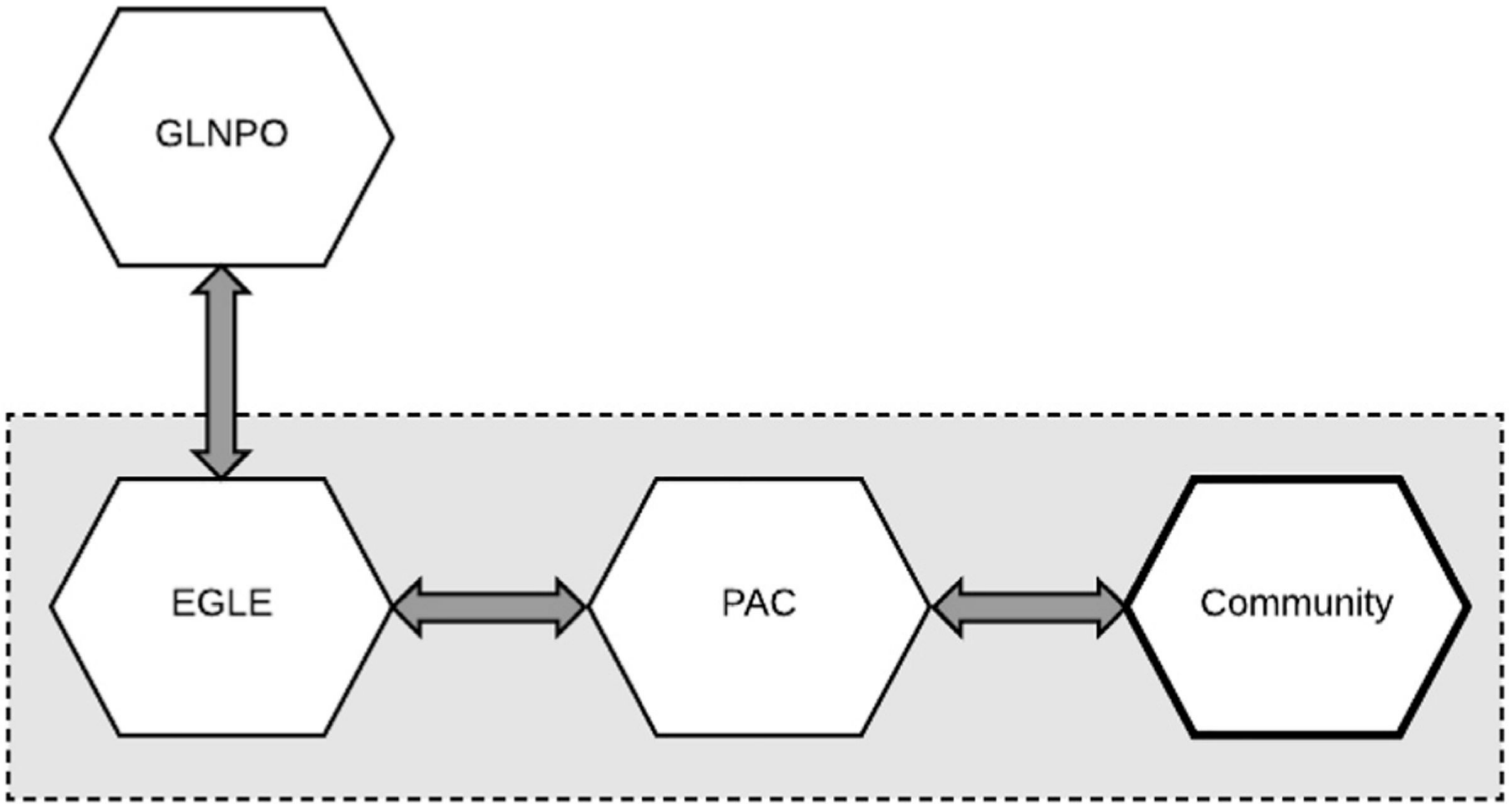
An organizational map of the primary entities in the Areas of Concern (AOC) program for our study areas. The gray area with the dashed line represents the programmatic and geographical extent of the AOC, in other words the ‘AOC space,’ and encompasses the perspectives gathered in this research. The line around the community entity is bolded because it is the primary focus of this study. The arrows denote which entities are related to each other and the directions of communication. Acronyms: GLNPO (Great Lakes National Program Office); EGLE (Michigan Department of Environment, Great Lakes, and Energy); PAC (Public Advisory Council).

The AOC program has been designed to incorporate humans as part of the ecosystem, which has led to the creation of Public Advisory Councils (PACs) for each AOC (Hartig, 1997). Throughout the Great Lakes, these groups assume various names (e.g., Citizen Advisory Group, Public Action Committee) but each provides a similar role within their AOC (Alsip et al., 2021). PACs are composed of experts and professionals from a wide variety of fields, representing a diversity of voices from the communities they serve as they work to restore local waterways. As state officials at the Michigan Department of Environment, Great Lakes, and Energy (EGLE) work to implement Remedial Action Plans (RAPs) to remove Beneficial Use Impairments (BUIs) for AOCs, PACs coordinate with the state agency to incorporate an array of perspectives into ecological management actions through connections to local organizations and the general public. These binational efforts are led, in the U.S., by the United States Environmental Protection Agency (USEPA), specifically through the Great Lakes National Program Office (GLNPO), carried out by state governments who oversee and direct progress in AOCs, and supported by PACs who advise cleanup efforts and management actions (Fig. 1).

For the AOC program to successfully implement an ecosystem-based approach, restoration must include collaborative efforts among stakeholders and bottom-up approaches to management (Hartig et al., 1998). Ecosystem-based management (EBM) examines complex environmental problems through a holistic ecosystem lens, considering all interrelationships and outcomes of all living things, including human use of resources (Hartig et al., 1998; Sproule-Jones, 2002). The complexities of protecting natural resources through EBM necessitates a host of different managers and jurisdictions (Brody, 2003) and requires a collective effort among the various groups involved (Selzer et al., 2020). Beierle and Konisky (2000) discussed benefits of stakeholder inclusion in the decision-making process within AOCs specifically, such as producing locally relevant solutions, reducing conflict among diverse groups, and building capacity to institute long-term positive impacts to environmental restoration. Given the unique nature of the impairments in each AOC, local voices are essential for implementing relevant solutions (Hartig et al., 1998).

While the creation of the AOC program represented an opportunity to apply EBM practices throughout the Great Lakes region, implementation of these bottom-up approaches has not been consistent across AOCs (Alsip et al., 2021; Beierle and Konisky, 2000; Hartig, 1997; Hartig et al., 1998; MacKenzie, 1993). Differences in management priorities across local, state, and national jurisdictions may result in complex environmental management scenarios (Brody, 2003). In practice, these complexities may mean that public participation and stakeholder inclusion vary among AOCs (Beierle and Konisky, 2000). Lack of a defined approach or direction can present difficulties for organizations in AOCs who lack resources to implement these strategies to effectively involve the public, including outreach expertise, organizational capacities, or power to influence. While the AOC program encourages stakeholder involvement through PACs, there still appears to be a disconnect in understanding between the restoration work completed by the program and the communities they serve (Holifield and Williams, 2019). Although there has been research of stakeholder involvement within the AOC program, an aspect that has been missing from previous research is the community or general public perspective, and their understanding of environmental progress over time. This research explores the community, PAC, and AOC program perspectives to better understand the connection and relationship between communities and the work of the AOC program.

This study seeks to better understand the complexities of community engagement among AOCs by comparing community use and views of local water resources to perspectives of PAC members and state officials on local water resources and community engagement opportunities. We investigated these perspectives through interviews, focus groups, and participatory observations in three Michigan AOCs. This study was conducted on behalf of EGLE by our team of three master’s degree students and two advisors at the University of Michigan in the School for Environment and Sustainability in fulfillment of the thesis requirement, and was compiled into a final report (Madden et al., 2020). Finally, we suggest potential solutions to enhance public engagement within the AOC program which may provide increased opportunities for communities to fully realize progress of local environmental restoration and benefits of the AOC program.

## Methods

### Case study AOCs

**Fig. 2.**
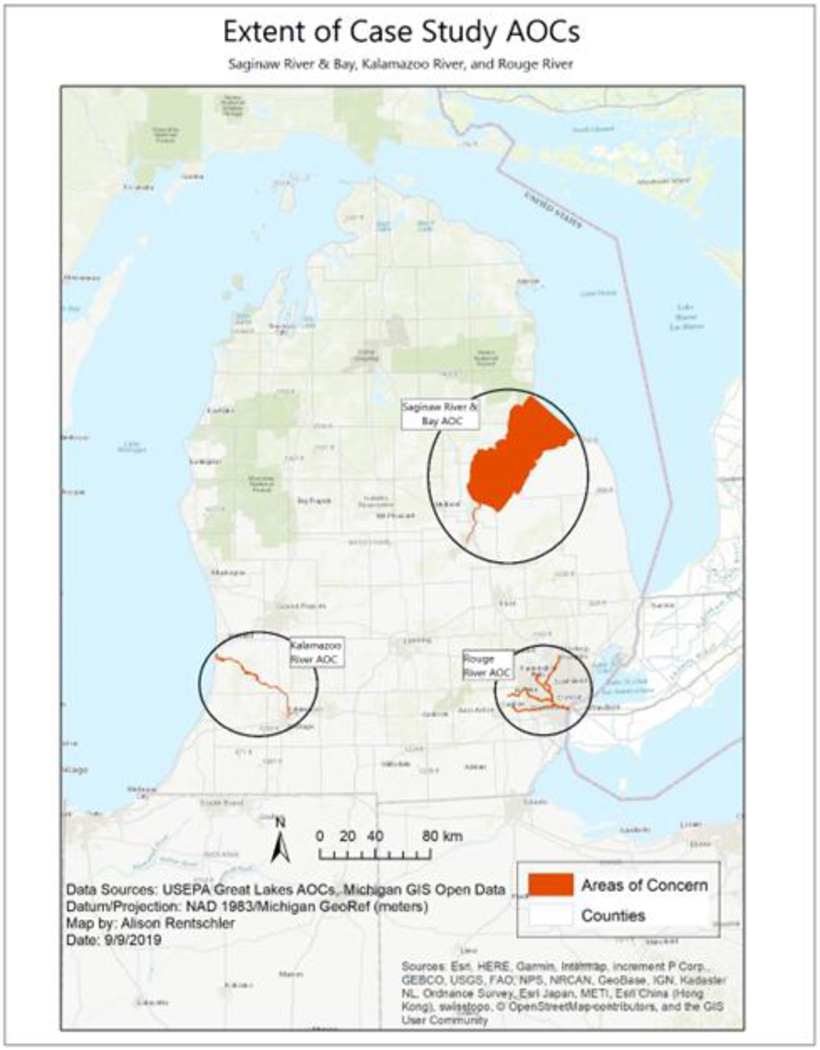
AOC map depicting the three case study locations: Saginaw River and Bay, Kalamazoo River, and Rouge River.

Our project team used a comparative case study approach to investigate community perspectives in three AOCs in the state of Michigan: the Kalamazoo River, the Saginaw River and Bay, and the Rouge River (Fig. 2). Comparative case studies are used to better understand how a phenomenon, such as the dynamics of community engagement in AOCs, works in context (Yin, 2009). These locations were chosen, in consultation with EGLE, because of their complex aquatic contamination, diverse landscape, and perceived slow progress towards removal of BUIs and delisting (Table 1). As broad stakeholder involvement allows for successful implementation of RAPs (Hartig, 1997), strengthening community engagement within these complex AOCs could encourage progress. We utilized data triangulation to allow for holistic investigation of AOC communities, employing three data types including participant observations, interviews, and focus groups.

### Participatory observations

Participatory observation research is a qualitative method where observers “learn about the activities of the people under study in the natural setting through observing and participating in those activities,” (Kawulich, 2005, pg. 2). Participant observation allows the researcher to gain an additional perspective into the worldview of the subject through the lens of personal experience.

In this case study, researchers conducted and compiled observations for three different settings: AOC access sites (e.g., parks, natural areas, beaches) (Electronic Supplementary Material (ESM) Figs. S1–S3), meetings (i.e., PAC and local organization meetings), and events (e.g., river clean-up days, educational hikes, paddling events) (ESM Table S1). We visited 45 access sites among the Kalamazoo (13 sites), Saginaw (17 sites) and Rouge (15 sites) AOCs and attended seven meetings and seven events among the three AOCs. Access site observations noted community interactions with the space with a focus on water resources, while meeting observations were taken at PAC or affiliated organization meetings to record agendas and sentiments, and event observations were taken at local community events aimed at connecting communities with their local water resources. Standardized templates were used for each observation type to allow for data comparison across AOCs (ESM Figs. S4–S6).

### Interviews and focus groups

Interview methods are commonly used to gain insight into “knowledge, values, beliefs or decision-making processes of stakeholders,” (Young et al., 2018, pg. 10). We used semi-structured interviews to allow for comparison across interviews while maintaining flexibility in conversation (Young et al., 2018). Interviews were conducted with individuals in different positions within the AOC program, including state-level EGLE officials, PAC members, and community members. Each interview lasted approximately-one hour, and was recorded and later transcribed. Interview questions encompassed perceptions related to roles at the individual and agency level, relationships between the AOC program and communities, and community connection and understanding of local water resources (ESM Figs. S7, S8).

In addition to semi-structured interviews, researchers used focus groups as a way to “listen and gather information” and to “better understand how people feel or think about an issue,” (Krueger and Casey, 2009, pg. 2). The focus groups ranged from four to 12 people as recommended by Krueger and Casey (2009), with four focus groups conducted among the AOCs (Table 2) (ESM Figure S9). Members of the focus groups were identified by reaching out to community groups via social media and email, as well as through local canvassing in parks, businesses, and events. Effort to gather the community perspective strictly through focus groups was affected by difficulties in recruiting participants. In cases where fewer than four individuals participated, we instead hosted smaller group conversations which we call ‘group interviews.’ We held four of these smaller group conversations. These interviews provided valuable insight into community values and perceptions and were thereby included in the data.

### Data analysis

We used a two-stage process of inductive and deductive qualitative data analysis in this comparative case study. We first applied inductive coding to interview and focus group transcripts, where major codes were distilled by extracting common themes from multiple interviews. This codebook was co-created through multiple iterations of coding exercises in order to ensure data reliability and consistency in the use of codes (ESM Table S2). Once the inductive codebook was created, all interviews and focus groups were coded through this process – assigning codes to quotes in order to organize and compile the data based on our research objective of understanding community engagement in AOCs.

To combine and analyze both interview and observation data into one framework, deductive coding was used through the lens of the Neighborhood Model (NM), which acts as a codebook designed to relate different perspectives through a place-based model (Williams et al., 2018). The NM allowed us to examine how different entities (i.e., individuals, organizations, and agencies) relate to a given space - in our case, how communities relate to the AOC neighborhood. There are four dimensions in the model that represent different neighborhood features including structural elements of a place (e.g., biophysical environment, infrastructure, governing rules), the built environment (e.g., schools, parks, housing), personal experiences (e.g., place attachment, desire to participate, feelings of safety), and human-environment interactions (e.g., aesthetics, sustainability) (ESM Figure S10). Analyzing the data with these dimensions helped to organize and identify what was important to different perspectives. For example, community members mentioned their personal experiences with a place (i.e., the personal experience dimensions), which is different from state agency officials who mentioned management actions to clean up a site (i.e., the structural dimension). Analyzing all data with this model allowed us to better understand the relationship between the AOC program and their communities from each perspective.

### Case study results

Three major themes that emerged from the data are described below. Results are presented for each of the case study AOCs, organized by these three themes. The three major themes that characterize each AOC include (1) community-resource relationship, (2) community-PAC and AOC relationship, and (3) strengthening the community-PAC and AOC relationship.

#### I. Community-Resource Relationship

This theme encompasses data describing the relationship between AOC communities and their local water resources, expounding on the ways in which communities use the resource, their understanding of environmental impairments, and their hopes for the future. Specific sub-themes within this section include (1) beneficial uses, (2) community perception of impairments, and (3) hopes for the future. The first two sub-themes relate to an AOC program concept of Beneficial Use Impairments, or BUIs, which are impairments to water bodies that negatively impact the ability to use the resource. Each AOC has a selected set of the list of 14 possible BUIs identified by the International Joint Commission (IJC) (IJC, 2012) (Table 3). BUIs are removed through remediation efforts and subsequent improvement of environmental condition, resulting in restored uses to water bodies. Because community members do not use program language, these concepts are described here as beneficial uses and impairments. The third sub-theme outlines community perspectives on hopes for the future of local water resources and points to motivations and values related to improving environmental quality.

#### II. Community-PAC and AOC Relationship

This theme exhibits data that describes the relationship between AOC communities and their local PAC or the AOC through efforts of community engagement. In some cases, the PAC is not the primary organization that engages the community to interact with or learn about their local water resources. In these cases, roles of relevant community organizations are also discussed. Additionally, because state and federal entities such as EGLE and GLNPO, respectively, are responsible for implementing the AOC program, specific state and federal program activities for each AOC, especially within the context of community engagement, is provided. Specific sub-themes in this section include (1) state and federal context, and (2) PAC roles and community engagement.

#### III. Strengthening the Community-PAC and AOC Relationship

In examining the connection between communities and the AOC or PAC, we recognized various contextual factors that appeared to be barriers to engagement in each AOC. These barriers were primarily attributed to a lack of resources and capacity to attract and sustain community participation, as described in PAC and state agency interviews. In spite of the barriers, there are opportunities to deepen the connection between AOC communities and their local PAC or AOC. This theme discusses ways to improve the connection between communities and the AOC program through creative partnerships with community-identified organizations.

### Kalamazoo River AOC

#### I. Community-Resource Relationship

##### Beneficial Uses

When discussing beneficial uses of the Kalamazoo River, community members most often mentioned canoeing or kayaking and fishing (Table 4). Community members also described improvement to aesthetics as well as a boost in the local economy through the revitalization of waterfronts. Community members throughout the Kalamazoo River watershed valued recreation, mentioning boating on local lakes and canoeing or kayaking on the river itself. Other community members discussed the importance of being able to walk along the river and enjoying the ability to take in its natural beauty.

Our team observed other recreational activities at sites including walking, hiking, relaxing and enjoying the scenery, and paddling. Many sites had built infrastructure for these activities (e.g., fishing docks and canoe launches). Another often discussed recreational beneficial use was beach-going. Three of the four most populated sites had beach access – Saugatuck Dunes State Park, Mount Baldhead Park/Oval Beach, and Markin Glen Park.

##### Community Perception of Impairments

Community members most often described impairments that are clearly visible or widely known, such as presence of “three-headed carps” or animal deformities, limited number of fish species, algae growth, and general sediment contamination (Table 4). Other impairments community members mentioned were topics of environmental concern, but outside the scope of the AOC program. These included flooding and PFAS (per- and polyfluoroalkyl substances) in drinking water, neither of which are directly connected to current BUIs. Community members also described conditions that were related to BUIs that have already been removed from the Kalamazoo AOC, which included degraded aesthetics (e.g., murky water, high turbidity, excess algae), and beach closings (inability to swim in the river).

Participants also mentioned concerns related to human health and quality of local water resources. One major health concern was polychlorinated biphenyls (PCBs) in the river due to industrial paper processing which led one interviewee to recall, “This river used to run multiple colors depending on what color crepe paper they were making in the paper mill that day.” Many community members described past negative experiences due to environmental contamination that have stayed with them their entire lives. One explained, “You could see so many dead carp [in Lake Allegan] that...you could literally walk across the surface.” Others recounted the severe impact of the Enbridge oil spill that occurred in 2010 near the AOC boundary. Because of the history of largescale environmental neglect caused by industry and the perceived inability of the government to enforce accountability, there was a general sense of mistrust from community members of government agencies.

##### Hopes for the Future

In spite of mistrust in government due to perception of culpability of environmental neglect, community members described hopes of learning more about and gaining a better understanding of their local environment. In particular, interviewees described a desire to know more about nature-based events and the current state of water quality in their area so they could foster a deeper connection to their environment and feel safe when recreating on the Kalamazoo River.

#### II. Community-PAC and AOC Relationship

##### State and Federal Context

State and federal activities and responsibilities in the Kalamazoo River AOC are related to the Superfund designation of the area. Because of heavy sediment contamination from PCBs in the Kalamazoo River, much of the responsibility for environmental remediation belongs to the federal government through the Superfund program and its ability to hold Potentially Responsible Parties (PRPs) accountable, according to both state agency and PAC member interviews. PAC and state officials would often discuss Superfund progress at PAC meetings and how this work greatly affects AOC progress. Others used the terms ‘Superfund’ and ‘AOC’ interchangeably. EGLE provides leadership for the AOC program, coordinating resources for AOCs to clean up contamination and ultimately remove BUIs and delist from the program. From EGLE interviews, officials describe themselves as liaisons between PAC organizations and the federal government, working to incorporate PAC input into clean-up efforts. EGLE officials assist the PAC in obtaining resources (e.g., consulting, funding), sustaining progress through efforts for BUI removals, and distributing funding from GLNPO for certain outreach events for the PAC.

##### PAC Roles and Community Engagement

The Kalamazoo River Watershed Council (KRWC) serves as the PAC for the Kalamazoo River AOC. Though the KRWC serves as the PAC, members described their primary role as the watershed council, with one PAC member noting, “Within five years [after the organization’s founding] being a PAC was not the most important function of the group.” The KRWC is the primary NGO that works to improve water quality throughout the Kalamazoo River watershed, educate the public on local contaminants, and host events to connect the public with their local water resources. As a community organization, the KRWC takes on community engagement through a host of educational mediums including signage, newsletters, events, and online resources. Despite the number of outreach methods, no community members interviewed specifically named the KRWC as an organization involved in improving the health of the watershed, although two respondents said they knew of this group when prompted. A quote from a community member kayaking at a local paddle event hosted by the KRWC exemplifies this lack of community awareness of the KRWC and the PAC, “We’ve been in Kalamazoo our whole life... and we didn’t know about [the KRWC]. How could we miss this, because we do love to do this.”

The primary outreach method that the KRWC uses to reach the community is local events. One popular event, as identified by meeting and event observations, is Kanoe the Kazoo, which is held multiple times per year and can attract 50–70 individuals each time. These paddling events provide community members an opportunity to recreate and connect with their water resources while learning about issues in their watershed. Additional KRWC educational efforts include informational campaigns related to ‘Do Not Eat the Fish’ signage, rain garden classes, and river cleanup events.

#### III. Strengthening the Community-PAC and AOC Relationship

##### Partnerships with Affiliated Organizations

In interviews, PAC members expressed wishing to better connect the “Kalamazoo River community” to their local resources and the AOC program. PAC members also lamented the difficulty of this task, limited resources, and their capacity. One PAC member noted, “...there’s a paid staff of one. I don’t think you can really achieve the level of engagement in the current broader socioeconomic environment with one person in that role.” In the past, the KRWC has partnered with universities including Western Michigan University and Michigan State University to educate the public on local environmental concerns. Community members identified these two universities, among others, as entities working to improve the health of the watershed. Additionally, interviewees identified several groups working towards a healthy watershed including local government entities such as the City of Kalamazoo and Kalamazoo Public Services, as well as watershed groups such as the Four-Township Water Resources Council.

### Saginaw River and Bay AOC

#### I. Community-Resource Relationship

##### Beneficial Uses

The most popular beneficial uses of the Saginaw River and Bay identified in community interviews were viewing the water, economic benefits (e.g., commercial fishing, tourism), boating (i.e., rowing, sailing, speed boats), fishing, and viewing wildlife (Table 5). Regarding the popularity of fishing, one community member noted, “There’s a lot of people fishing on the river, I’m seeing all kinds of generations of kids, adults, older kids, fishing.” Other beneficial uses that community members described in interviews included paddling, ice fishing, walking and hiking, and biking. Our observation data indicated that beaches had the highest visitation, as compared to rivers or wetlands in the AOC. Activities that were most often observed at sites included walking and hiking, beach-going, fishing, and viewing wildlife. The top three activities included beach-going, fishing, and walking and hiking (Table 5).

A primary beneficial use of the Saginaw River and Bay, as described by both PAC and community members is the aesthetic value. This is corroborated by site observations in which community members enjoyed recreational opportunities that allow for viewing of nature and water resources, including beach-going and hiking or walking. Fishing and boating were also confirmed as important by observation data as 15 of the 17 sites included built infrastructure to allow for fishing (e.g., boat docks and piers) (Table 4). One individual spoke with us at an access site and explained their value in being able to fish, noting, “I catch fish here often with my sister.”

##### Community Perception of Impairments

The community identified several water quality impairments, including fewer fish, polluted sediments, runoff from industry and agriculture, and degraded aesthetics. These impairments as described by community members align with BUIs defined in the GLWQA. Community and PAC members described other impairments that are not included in the GLWQA including fish consumption advisories, flooding, and invasive species in interviews and focus groups (Table 5).

While community members described enjoyment of natural spaces and valued these spaces, many still expressed concern about and described a stigma associated with the quality of water resources based on both historic and current quality of the resource. One community member noted, “...it’s really too bad because everybody in this area knows how dangerous...how polluted the water is here.” Multiple people remembered Saginaw Bay’s lack of winter ice because of industrial contamination, sharing, “The 40s and 50s - when the Saginaw River was generally recognized as the most convenient place to put your sewage.” Other individuals recalled the lack of bald eagles among riparian corridors. Because of these memories, some still believe that the Saginaw River, specifically, is still highly contaminated.

##### Hopes for the Future

Despite pervasive negative perceptions, community members voiced a hope for an improved environment for their family and for future generations. One community member described the desire to maintain environmental quality so his child may be able to experience the beauty of these natural spaces as they grow up, “I hope that we don’t damage the things that he might not be able to see when he grows up.” Another individual expressed their desire to be in nature to view wildlife and deepen their notions of being part of an ecosystem. These concepts of sustainability and familial ties draw the community to visit these natural spaces.

#### II. Community-PAC and AOC Relationship

##### State and Federal Context

In the Saginaw River and Bay AOC, interview and meeting observation data confirms that EGLE is the primary entity coordinating “federal, state, and local resources to clean up some of the state’s most heavily impacted, contaminated areas” as one state official described. EGLE additionally provides guidance to PACs by attending PAC meetings. Aside from EGLE, the Michigan Department of Natural Resources (MDNR) has a major role within this AOC, providing public access to the Saginaw River and Bay as the landowners of boat launches, protected wildlife areas, and state parks.

##### PAC Roles and Community Engagement

The Partnership for the Saginaw Bay Watershed (PSBW) serves as the PAC for the Saginaw River and Bay AOC. PAC members described the duties of the PSBW as project planning, providing a voice for concerns of the community, and working toward delisting the Saginaw River and Bay as an AOC. One PAC member described their role as, “prioritizing projects and problems that need to be solved and taken care of, coming up with plans that need to be implemented...all leading toward cleanup and eventual delisting of the Area of Concern.” While most members agree on PAC roles related to project planning and prioritizing BUIs, as a group they do not agree on their responsibility for public outreach and stakeholder inclusion. Resource constraints, particularly monetary resources, are noted as a barrier to both progress and public engagement, with a PAC member stating, “We don’t have the big money push behind us and yet we’re the ones with the biggest, most expensive problems.”

Some PAC members expressed that public outreach should be a primary objective while others believe that resources are better spent elsewhere. These diverging views on outreach and a lack of resources led to the PSBW conducting minimal community engagement: “Our communication with the PAC back to local industry, local residents, and local government is very limited.” This is corroborated by the community data in which no community members were able to identify the PSBW as an entity working on water quality in the Saginaw Bay area. Some PAC members desire increased connection to community groups, with one PAC member saying at a meeting, “There’s a lot of potential partners - let’s live up to our name.” Though the PSBW does not directly engage with the public, community ties can be seen through PAC membership, including Little Forks Conservancy, Bay County, and the Saginaw Chippewa Indian Tribe of Michigan.

#### III. Strengthening the Community-PAC and AOC Relationship

##### Partnerships with Affiliated Organizations

There are a number of community organizations throughout the Saginaw Bay watershed that work to engage the public. As compiled throughout all data types, these groups include land conservancies, tribal nations, recreational outfitters, universities, and industry. Organizations such as these have a significant role in educating and engaging the public through volunteerism, recreation, and conservation efforts. Select examples of such community events include the Little Forks Conservancy’s invasive species hikes and the Tall Ships Celebration in Bay City sponsored by a multitude of local businesses, news outlets, and universities. Using site observation data, we found community groups also provide access to the Saginaw Bay or River by constructing boat launches or conserving vital ecosystems for public use. Through creative partnerships, the PSBW could further leverage relationships to these community groups to allow for a deeper connection between the Saginaw Bay area community and the AOC program.

### Rouge River AOC

#### I. Community-Resource Relationship

##### Beneficial Uses

The beneficial uses most often mentioned by the community in interviews included viewing wildlife and nature, fishing, and canoeing or kayaking (Table 6). Other beneficial uses that the community described included economic benefits of tourism or increased real estate values, and recreational activities such as swimming, biking, and hiking. One community member identified the importance of the diversity of beneficial uses of the Rouge River, “Heinz Park [is] used very heavily for all kinds of things both water-related and not, but I think there are a lot of people that appreciate nature and like to hike along the river.”

From our observational data, the most popular sites with more than 100 visitors were Heritage Park in Canton and Heritage Park in Farmington Hills. Both of these locations provided outdoor space for families to recreate in green spaces with amenities such as play structures and splash parks. Common activities recorded at other sites included walking or hiking, cooking and eating, and viewing nature. Other activities recorded at observation sites in the Rouge River AOC included playing, dog walking, exercising, fishing, socializing, relaxing, and participating in environmental restoration efforts.

##### Community Perception of Impairments

This community’s comments about environmental impairments in the AOC were directly related to aesthetics. Community members describe the Rouge River as “junky,” “dirty,” and having a foul odor. Participants also described flooding given the extensive amount of impervious surfaces (Table 6). While specific contaminants were not mentioned, there was a general consensus that the river, primarily in the past, was not healthy and potentially dangerous, with some lingering perceptions of current environmental degradation.

In the Rouge River AOC, community members have a negative stigma attached to the river because of the poor water quality resulting from a long history of industrialization and urbanization. Focus group participants relayed their experiences related to this negative stigma, recalling that the Rouge caught fire because of heavy industrial contamination. One community member further explained, “When I was a kid in the 60s, sometimes we couldn’t even go outside because the stink from the river was so bad. We were told we couldn’t wade or even touch the water because it was so full of toxins.” Community members still have an aversion to the river today because of these memories, especially avoiding areas near the industrial section where quality is more impaired. For others, there is a sense of pride in how far the Rouge River has come since restoration efforts began in earnest, particularly since the environmental movement of the 1970’s and the establishment of the EPA. Community members are especially proud of their role in clean-up efforts through river restoration events and rain garden plantings.

##### Hopes for the Future

Rouge River community members described hope for a future in which the river is a celebrated focal point. One community member expressed this vision through the example, “...to me it would be that the river is part of the community. Like a street is part of the community or a park or a movie theater or something like that. You think of that as, it is part of you and you are part of it.” Focus group members also often discussed the importance of instilling environmental values in youth and future generations. While some participants discussed a lack of youth interest in natural spaces, others described leading citizen science groups to educate kids on water quality or volunteering to clean up the river in order to help foster environmental values among youth.

#### II. Community-PAC and AOC Relationship

##### State and Federal Context

GLNPO has brought important partnerships to the table in the Rouge River watershed according to interviewees, who provided the example of the inclusion of Honeywell International Inc. as a major funder for various projects to remove contaminated sediment through the Great Lakes Legacy Act. The Great Lakes Legacy Act was established in 2002 to provide federal funds for sediment remediation projects in Great Lakes AOCs (Alsip et al., 2021). PAC and state agency representatives mentioned this as an important aspect that affects progress in the AOC. In the Rouge River, PAC and agency interviews indicated that EGLE is responsible for producing restoration action plans in conjunction with the federal government, providing communication on expectations from the federal and state government, and securing funding and resources for the PAC.

##### PAC Roles and Community Engagement

The Rouge River PAC is the Rouge River Advisory Council (RRAC). The RRAC is housed within the Alliance of Rouge Communities (ARC), a non-profit organization of municipal governments and partnering groups with a mission to meet regional stormwater permits. ARC helps to set up and run RRAC meetings and apply for grants, utilizing their status as a 501(c)(3) nonprofit organization. PAC and state representatives primarily described RRAC roles of project planning, advising EGLE on decisions related to BUI criteria and removal, and prioritizing impairments and project locations within the watershed. Interviewees described RRAC as a technical organization that did not currently have community outreach functions. When asked about the PAC’s engagement with the public, one PAC member said “I’m not sure that there is a relationship” but that community representatives on ARC provide a connection. Interviewees did not describe community engagement as a primary focus of this organization. In fact, community members did not identify RRAC as an organization that worked on water quality in the region. However, RRAC did previously have an outreach committee but this has since been dissolved due to lack of funding and resources, according to a PAC interviewee.

The primary community-facing organization in the Rouge River AOC is the Friends of the Rouge (FOTR). This watershed council “operates in many ways as the gateway to stewardship for citizens within the watershed,” as one interviewee described, providing unique ways for community members to interact with their local water resources. FOTR reaches the Rouge community through river clean-up events, fish and macroinvertebrate monitoring programs, and partnerships with local organizations. Stewardship is an important aspect of FOTR’s mission. As a result, they organize events to encourage volunteers to participate in restoration in several sections of Rouge River watershed. These activities foster environmental values and deepen connections to the river. Three of the events attended by researchers within the watershed included aspects of active stewardship and restoration, including two rain garden install events and a river cleanup based around converting trash found in the river into an art installation. Between FOTR and RRAC, there is overlap in membership which has allowed for a coordination of resources. This coordination is exemplified through EGLE-funded FOTR fish monitoring through previous PAC grants.

#### III. Strengthening the Community-AOC Relationship

##### Partnerships with Affiliated Organizations

It is clear that FOTR has a strong relationship to the Rouge River community through their extensive stewardship efforts. In addition, FOTR has a functional working relationship with RRAC through overlap in membership and coordination of resources. Community members additionally mentioned working with other community organizations to restore the Rouge River, including the Girl Scouts of America, the Boy Scouts of America, the Detroit Zoo, River Restorations, Friends of the Detroit River, as well as various local garden clubs. By focusing partnership efforts with these community organizations, RRAC and the AOC program can work to improve understanding of and engagement in restoration efforts among community members.

## Discussion

### PACs and community engagement

**Fig. 3.**
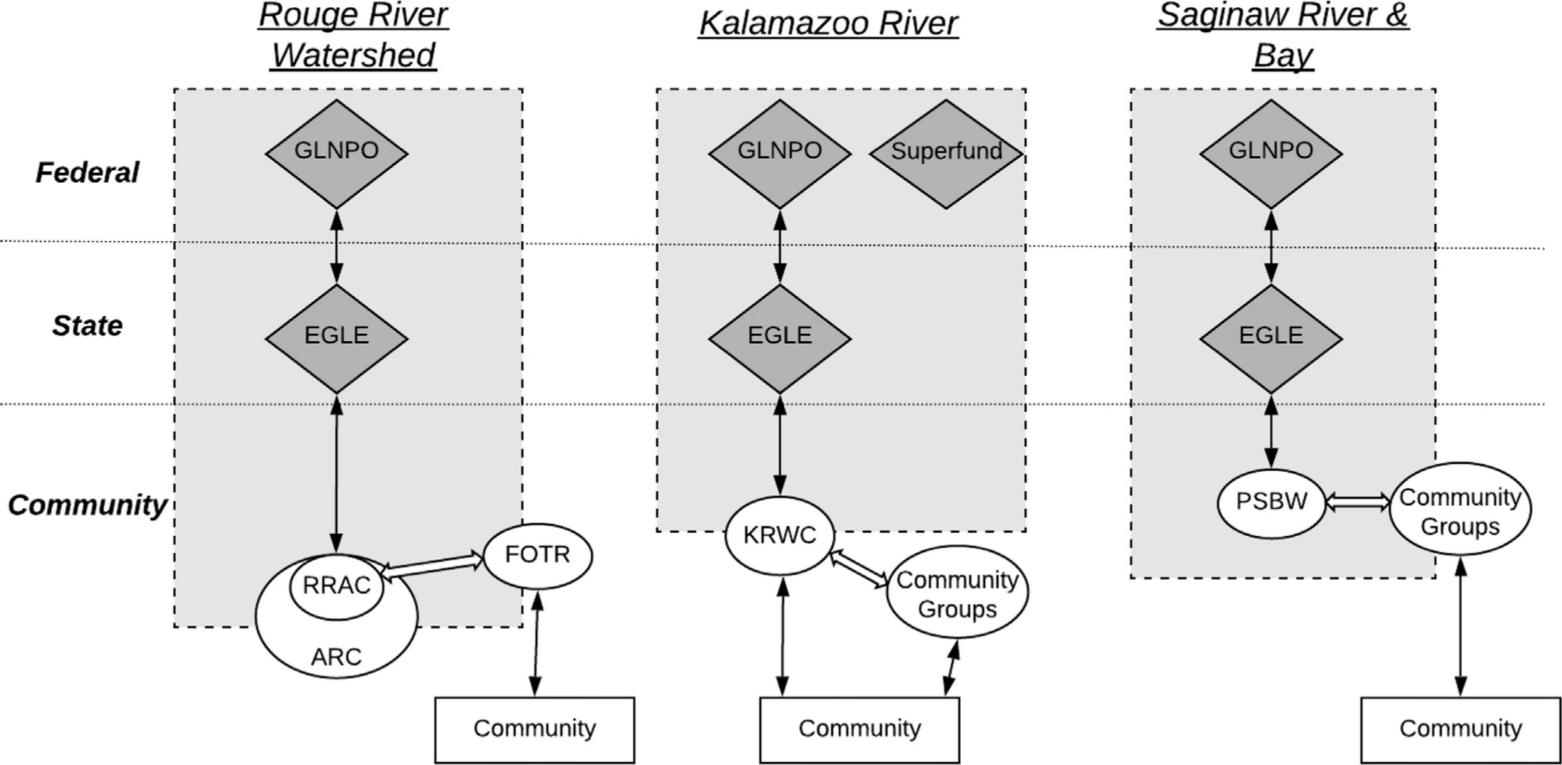
Relationship diagram of each case study AOC. The purpose of this diagram is to visualize relationships within the AOC program in each case study AOC, and to better understand which entities fit squarely within the ‘AOC space’ and which entities conduct work outside of the AOC space or entities that are unaware of the program as a whole. Relationship in this context denotes two groups that interact with one another through communication or other social or governmental ties. The gray area with the dashed outline represents the AOC space. Dark gray rhombuses represent governmental organizations, and ovals represent non-profit or non-governmental organizations. The solid bi-directional arrow indicates a direct relationship between entities. A white arrow represents a relationship among organizations in which there is member-sharing. The concentric circles of RRAC (Rouge River Advisory Council) and ARC (Alliance of Rouge Communities) indicate that the RRAC is house within the organization ARC. Additional acronyms: GLNPO (Great Lakes National Program Office); EGLE (Michigan Department of Environment, Great Lakes, and Energy); FOTR (Friends of the Rouge); KRWC (Kalamazoo River Watershed Council); PSBW (Partnership for the Saginaw Bay Watershed).

Through analyzing interview data among community members across case study AOCs, it was clear that the majority of individuals were unable to identify their local PAC as an entity working to restore water resources, nor identify their community as an AOC (Fig. 3). This may reflect the observation that the PACs studied tended to engage in activities such as project planning and prioritizing BUIs, rather than engaging the community at large. The major exception to this is the KRWC who see themselves primarily as a watershed council, focusing on educating individuals on water quality and resource management, in addition to serving as the PAC for the Kalamazoo River AOC. Despite this, most community members were also unaware of the KRWC and its activities within the watershed.

Our study suggests that public engagement is not a priority among case study PACs. Some PAC members questioned whether community engagement was within their responsibilities because of budget constraints and limited capacity. It appears that EGLE does not provide specific guidance for PACs to include community involvement as part of their duties, thus engagement is primarily left to the discretion of PACs themselves. There are some instances in which EGLE has funded community engagement in AOCs (e.g., Kanoe the Kazoo events hosted by the KRWC). However, the majority of AOC funding and effort is directed towards restoration projects and planning. As a result, community engagement may be absent or less robust in PACs that do not have the finances or capacity to take this on as a separate duty.

Our data illustrated ways that communities enjoy their environment, suggesting that individuals have an attachment to their environment and use valued spaces for activities. Further, while each of the case study AOC communities expressed lingering negative stigmas and hesitance to interact with their historically degraded water resources, they also recognized many positive attributes of their local water resources. Lertzman (2015) argues that perceived apathy towards engaging with the environment stemming from contamination is actually part of a larger psychosocial process of ‘environmental melancholia,’ relating to the loss of something loved. This deep community grief within AOCs exemplifies the great value that is placed on local natural resources. Community members across case study AOCs also stated desires to know more about their environment and the status of restoration. Increased community engagement through PACs or other local organizations could provide an important opportunity for community members to learn more about restoration efforts and foster a deeper connection to their environment.

### Community-level relationships through beneficial uses, impairments, and trust

While community members of case study AOCs do not tend to interact directly with PACs, they do tend to have strong connections to other groups within their communities (Fig. 3). Interview data provided insight into which organizations (e.g., watershed councils, universities) community members recognize as invested in water resource restoration (Table 7). Communities have strong relationships with these groups because these local organizations host events that reflect community-defined beneficial uses and impairments. In other words, these organizations are highly attuned to the values of the community which allow them to best connect with the public. For example, in the Rouge River AOC, the watershed council Friends of the Rouge provides both active opportunities like river clean-ups and monitoring events, and other less active events like the Rouge Cruise tour, which reflects the diversity in beneficial uses in the Rouge River community. Friends of the Rouge is also attuned to the community’s understanding of aesthetic impairments, hosting restoration events which allow the community to aid in direct environmental remediation. Through these events, local organizations are able to connect to community members in ways they care about and understand, strengthening the connection between AOC communities and their local water resources.

Because of their unique connection to communities, local organizations are often highly trusted sources for information and events. Some community members among case study AOCs expressed a lack of trust in governmental agencies’ ability to hold polluting industries responsible for environmental damage. Local organizations are removed from this negative association which allows community members to trust these groups to provide education, events, and spaces to recreate safely.

### Partnership for community engagement

Stakeholder involvement is a well-documented measure of success for ecosystem-based management and has been used as such throughout the Great Lakes AOC program. According to Alsip et al. (2021), integrative partnerships and broad-based stakeholder inclusion have proved successful among many AOCs to improve institutional capacities, funding, and progress over time. Our research further points to the merit of diversifying partnerships with stakeholder groups as these are the primary groups that engage directly with the general public and engage communities with their local resources. Our team has demonstrated that both community members and PACs currently have meaningful connections to local organizations (Table 7). In each of the case study AOCs, PACs consist of representatives from local groups that already have connections to the public. Examples include university representation on the KRWC in Kalamazoo, or non-profit representation through FOTR and Little Forks Conservancy for the Rouge River and Saginaw River and Bay, respectively. With increased dissemination of AOC information through existing channels, PACs can enhance capacity for community engagement so community members can learn about AOC work through organizations they already know and trust.

Research indicates that the formalization of strategic partnerships between community organizations and PACs could maintain ongoing and equitable community engagement (Garcia et al., 2021). Official partnership arrangements could become part of the AOC program through organizational invitations to join a PAC, or through the inclusion of community organizations on PAC outreach committees. One example from our research could provide a model for these kinds of relationships. Friends of the Rouge (FOTR) in the Rouge River AOC has cultivated a deep connection to community members by giving the community a role in restoration and opportunities for participation. This strong community relationship combined with FOTR’s connections to the Rouge River AOC PAC (RRAC) through overlap in membership creates an opportunity for increased engagement in the AOC. A similar partnership arrangement within the Great Lakes AOC program can be found in the Buffalo River AOC through the efforts of Buffalo Niagara Waterkeeper (BNW). BNW is a multi-faceted organization that houses the Remedial Advisory Committee for the AOC and catalyzes public–private partnerships to remediate and restore the Buffalo River. Their efforts connect community members to the Buffalo River by improving public access sites, hosting volunteer events, and creating environmental programs. Funding and projects implemented by entities such as the Federal Highway Administration, the New York Power Authority, the New York State Parks Department, and the City of Buffalo have produced significant progress to restore the Buffalo River as a pillar of the community (Jedlicka and Hartig, 2018). Partnership arrangements such as these can solidify relationships for long-term successes not only in public engagement, but also for overall funding, community capacity, and progress for the AOC program.

## Conclusion

The research presented in this paper provided evidence that community members in the Kalamazoo River, Saginaw River and Bay, and Rouge River AOCs have minimal knowledge of the AOC program and the remediation and restoration work that is being conducted to restore these areas. Not only were most respondents unable to identify their area as an AOC, they were also not able to identify their local PAC as an entity working towards environmental restoration. Given this lack of recognition, PACs among these complex AOCs may need assistance if they wish to improve the program’s relationship with communities (Fig. 1, Fig. 3). This is important because it is clear that each AOC community presented in this research cares deeply for their local environment, as displayed through described beneficial uses and hopes for the future. While community members may not need to know the specific workings of the AOC program, community members could benefit from a greater understanding of restoration efforts that directly impact their quality of life and attachment to sense of place.

We provide several recommendations to improve community engagement, primarily through intentional partnerships between PACs and local organizations that communities value and recognize. State agency facilitation of partnerships with regional planning committees or other capacity building organizations can help to reduce the burden on PACs to identify and build these relationships from the ground up. This challenge might be impossible for PACs with limited resources. This effort is needed because community engagement is critical for the public to understand progress toward restoring their AOCs, and to further foster a cohesive sense of place and local environmental and community pride.

Each of the case study AOCs demonstrated potential for the kinds of future partnership opportunities that can improve community capacity and boost public engagement within the AOC program. Systematizing partnership arrangements could create the conditions for greater dissemination of AOC information through varying channels to better engage the community in the AOC program. To better understand how to more effectively implement partnership arrangements in AOCs, future research should be pursued to understand community perspectives, as well as PAC and agency activities, in AOCs throughout the Great Lakes region to test our findings and build transferrable models. Following this, further research could also potentially quantify community engagement among AOCs and investigate how a larger conceptualization of beneficial uses and impairment could facilitate efforts towards delisting.

## Supplementary Material

SI

## Figures and Tables

**Table 1:** Descriptions of the three Areas of Concern of interest: the Kalamazoo River, the Rouge River watershed, and the Saginaw River and Bay. Information on the source of impairments comes from each AOC’s most recent RAP (Kalamazoo: Riley, 2012; Saginaw River and Bay: Selzer, 2012; and Rouge River: Selzer, 2008).

	Geographic Extent of AOC	Landscape Type	Population Density	Primary Pollutants or Concerns	Sources of Impairments	PAC

**Kalamazoo River**	An 80-mile stretch of the Kalamazoo River (including riparian areas) from Morrow Dam to the outlet at Lake Michigan Additional 3-mile stretch of Portage Creek in Kalamazoo, MI	Mix of rural and urban with ~ 50 % agricultural land^1^	Primarily low population density with pockets of mid-density in cities (Kalamazoo, MI density – 1,140 people per km^2^)	Polychlorinated Biphenyls (PCBs) in sediments; Excess nutrient load; Failing dams	Legacy contamination from paper processing; Agricultural runoff; Aging infrastructure	Kalamazoo River Watershed Council (KRWC)
**Saginaw River & Bay**	Entirety of Saginaw Bay, ending at an imaginary line drawn between Au Sable Point and Pointe Aux Barques Additional 22-mile stretch of the Saginaw River	Saginaw Bay Watershed is 45 % agricultural land with mixed wetland/forest areas and < 5 % developed regions^2^	Primarily low-density towns surrounding Saginaw Bay with highest density in Saginaw (~1,100 people per km^2^)	PCBs, dioxins, legacy sediment contamination; Excess nutrient and sediment load; High bacteria	Industrial contamination; Agricultural runoff Combined Sewer Overflows (CSOs) and point- and non-point source pollution	Partnership for the Saginaw Bay Watershed (PSBW)
**Rouge River**	Main river branches and tributaries contained within the Rouge River watershed	Primarily urban with 84 % developed land^3^	Highly populated watershed with an average of 1,100 people per km^2^ and a total of 1.3 million residents^3^	Urban stormwater runoff; PCBs, PAHs, oil, legacy sediment contamination; High bacteria	Impervious surfaces; Legacy industrial contamination; Combined Sewer Overflows (CSOs) and point- and non-point source pollution	Rouge River Advisory Council (RRAC)

Sources:

1 Alexander et al., 2014

2 TNC, 2018

3 Napieralski et al., 2015.

**Table 2:** Summarized PAC and community member interviews, focus groups, and group interviews in each AOC. Interviews were conducted with state officials to understand their perspective, but are not included in this table to protect anonymity.

	*Perspective*	Kalamazoo	Saginaw	Rouge	TOTAL

Interviews	*PAC*	3	4	3	**10**
	*Community*	1	1	2	**4**
Focus groups (4–12 people)	*Community*	1	1	2	**4**
Group interviews (<4 people)	*Community*	3	1	–	**4**
	TOTAL	**8**	**7**	**7**	**22**

**Table 3:** Past and current BUIs in each of the case study AOCs (USEPA, 2020).

BUIs	Kalamazoo	Saginaw	Rouge

Restrictions on Fish and Wildlife Consumption	X	X	X
Tainting of Fish and Wildlife Flavor		*Removed 2008*	
Degraded Fish and Wildlife Populations	X	X	X
Fish Tumors or Other Deformities			X
Bird or Animal Deformities or Reproductive Problems	X	X	
Degradation of Benthos	X	X	X
Restrictions on Dredging Activities	X	X	X
Eutrophication or Undesirable Algae		X	X
Restrictions on Drinking Water Consumption or Taste and Odor Problems		*Removed 2008*	
Beach Closings	*Removed 2011*	X	X
Degradation of Aesthetics	*Removed 2012*	X	X
Added Costs to Agriculture or Industry Degradation of Phytoplankton and Zooplankton Populations		X	
Loss of Fish and Wildlife Populations	X	*Removed 2014*	X
**TOTAL REMAINING BUIs**	**6**	**9**	**9**

**Table 4:** Community-defined beneficial uses and impairments for the Kalamazoo River AOC. Observation data comes from AOC access sites (e.g., parks, boat launches, nature preserves). Underlined impairments denote direct reflection of a defined BUI for the Kalamazoo River AOC.

Kalamazoo River AOC					
	Community-Defined Beneficial Uses				Community-Defined Impairments
	*Interviews*	*Observations*			*Interviews*
**Most common**	Kayaking & Canoeing Fishing Aesthetic Beauty Economic Benefits	*Most observed*	*Most participants*	**Impairments that directly impact most common uses**	Animal Deformities Degraded Aesthetics * Limited Fish Species Contaminated Sediment
		Walking & Hiking Viewing Nature Fishing Relaxing	Beach-going Walking & Hiking Kayaking & Canoeing		
**Less common**	Eating the Fish Swimming	Playing Picnicking Running Walking dogs Biking Feeding Fish Boating Socializing		**Other impairments**	Inability to Eat the Fish Invasive Species No Swimming* PFAS (Potable Water Quality) High Turbiditv* Urban Flooding

* BUI has been removed but is still perceived by the community as an impairment.

**Table 5:** Community-defined beneficial uses and impairments for the Saginaw River & Bay AOC. Observation data comes from AOC access sites (e.g., parks, boat launches, nature preserves). Underlined impairments denote direct reflection of a defined BUI for the Saginaw River and Bay AOC.

Saginaw River & AOC					
	Community-Defined Beneficial Uses				Community-Defined Impairments
	*Interviews*	*Observations*			*Interviews*
**Most common**	Viewing the Water Viewing Wildlife Economic Benefit Fishing Boating	Most observed	Most participants	**Impairments that directly impact most common uses**	Contaminated Sediment Reduced Fisheries Agricultural Runoff Excess Aleae Bad Odor
		Walking & Hiking Beach-going Fishing Biking Picnicking Viewing Wildlife	Beach-going Fishing Walking & Hiking		
**Less common**	Canoeing & Kayaking Ice Fishing Eating the Fish Drinking Water Commercial Fishing Biking Walking & Hiking	Playing Picnicking Running Walking dogs Biking Feeding Fish Boating Socializing		**Other impairments**	Restrictions on Fish Consumption Erosion & Sedimentation Flooding Invasive Species

**Table 6:** Community-defined beneficial uses and impairments for the Rouge River AOC. Observation data comes from AOC access sites (e.g., parks, boat launches, nature preserves). Underlined impairments denote direct reflection to a defined BUI for the Rouge River AOC.

Rouge River AOC					
	Community-Defined Beneficial Uses				Community-Defined Impairments
	*Interviews*	*Observations*			*Interviews*
**Most common**	Viewing Nature Fishing Canoeing & Kayaking	Most observed	Most participants	**Impairments that directly impact most common uses**	Dirtv Water Foul Odor Refuse in the River Log Jams Flooding
		Walking & Hiking Eating or Picnicking Viewing Nature	Playing on Structures Playing Sports Relaxing		
**Less common**	Tourism Real Estate Swimming Clean Water Biking Hiking	Exercising Dog Walking Fishing Socializing Relaxing River Restoration		**Other impairments**	Erosion Excess Runoff Impervious Surfaces

**Table 7:** Organizations participating in each case study AOC and organizations that were recognized by community members. Bolded text denotes the organizations most often cited by community members. The category ‘event observation host groups’ specifies community groups who hosted engagement events at which researchers observed.

AOCs	Total groups from all data	Groups from community perspective	
Kalamazoo River	● University groups (5) ● Native American tribes (1) ● Local businesses or consulting firms (4) ● Community groups and NGOs (19) ● Large corporations (2) ● Municipalities and county government (7) ● State and federal agencies and actors (10)	Interviews – key players	● **Universities (5)** ● Local businesses or consulting firms (1) ● **Municipalities and county government (4)** ● State and federal agencies and actors (3)
		Event observation host groups	● Nonprofit (1): Kalamazoo River Watershed Council
Saginaw River and Bay	● University groups (6) ● Funding organizations (1) ● Local businesses and consulting firms (9) ● Community groups and NGOs (25) ● Large corporations (12) ● Municipalities and county government (24) ● State and federal agencies and actors (9)	Interviews – key players	● Universities (4) ● Native American tribes (1) ● Local businesses or consulting firms (1) ● **Community groups and NGOs (15**) ● Large corporations (2) ● **Municipalities and county government (12)** ● State and federal agencies and actors (4)
		Event observation host groups	● Nonprofit (2): Little Forks Conservancy, Friends of the Shiawassee
Rouge River	● University groups (7) ● Native American tribes (1) ● Funding organizations (3) ● Local businesses and consulting firms (12) ● Community groups and NGOs (36) ● Large corporations (6) ● Municipalities and county government (24) ● State and federal agencies and actors (10)	Interviews – key players	● Universities (1) ● Local businesses or consulting firms (1) ● **Community groups and NGOs (4)** ● State and federal agencies and actors (1)
		Event observation host groups	● Nonprofit (2): **Friends of the Rouge**, Friends of Eliza Howell Park
