# Supplementary material for: Community engagement and the importance of partnerships within the Great Lakes Areas of Concern program: A mixed-methods case study
Source: J Great Lakes Res. Author manuscript; Available in PMC 2024 Jan 24. (PMC10807300; doi:10.1016/j.jglr.2022.08.005)

Figure S1. Kalamazoo Site Observations and AOC Extent Map


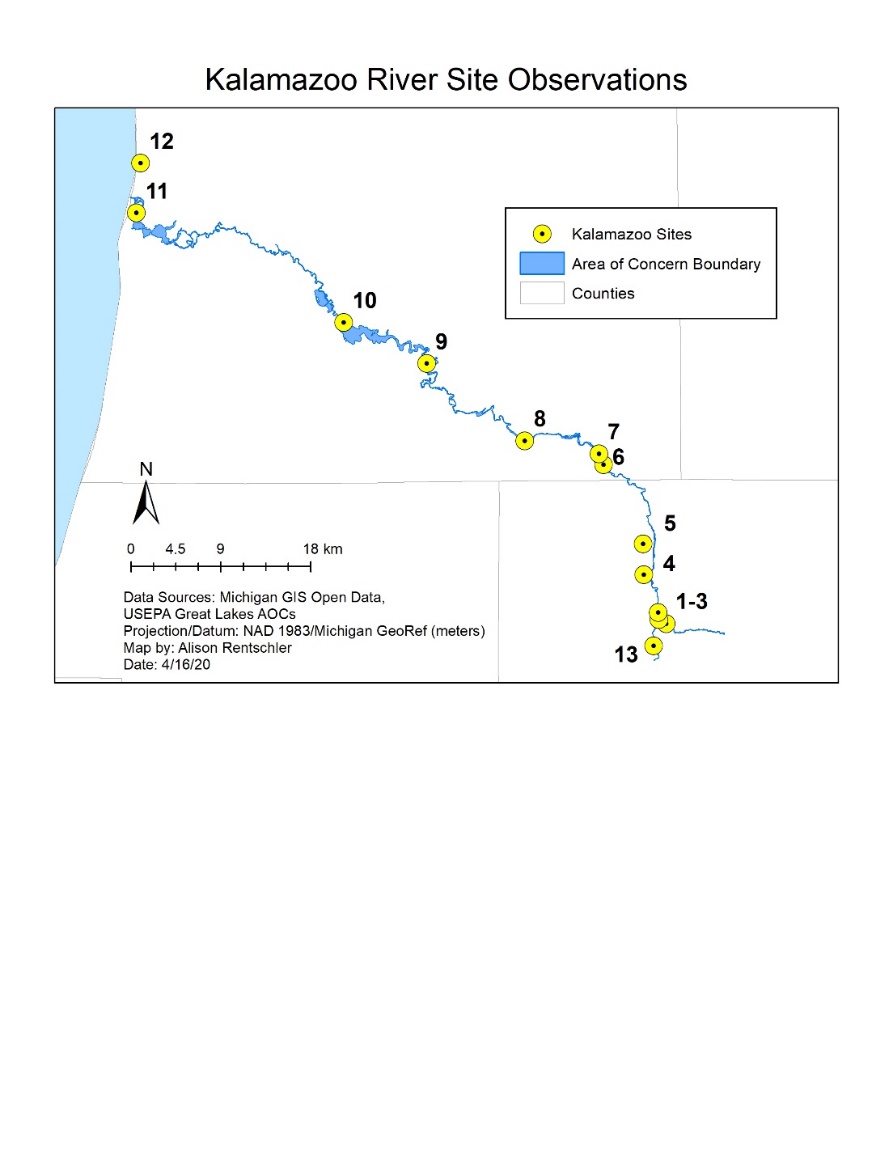

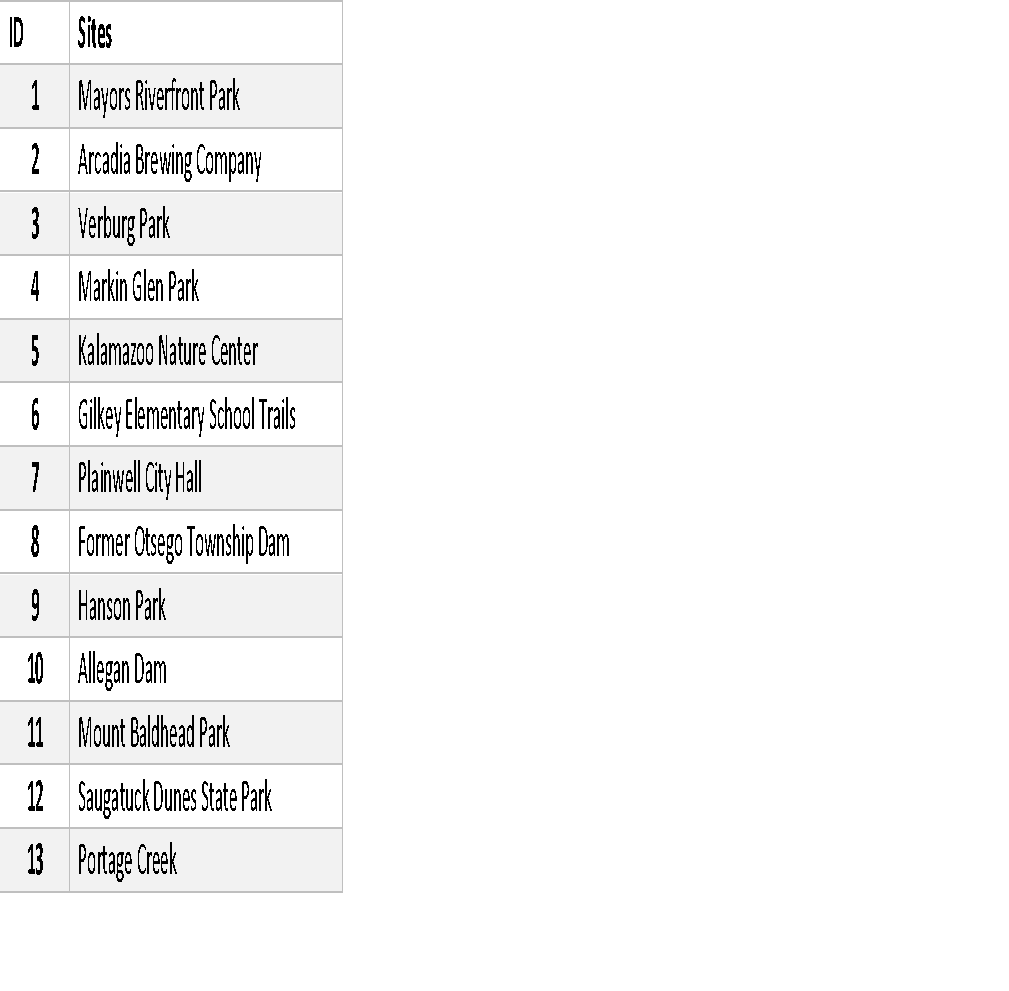


Figure S2. Saginaw River & Bay Site Observations and AOC Extent Map


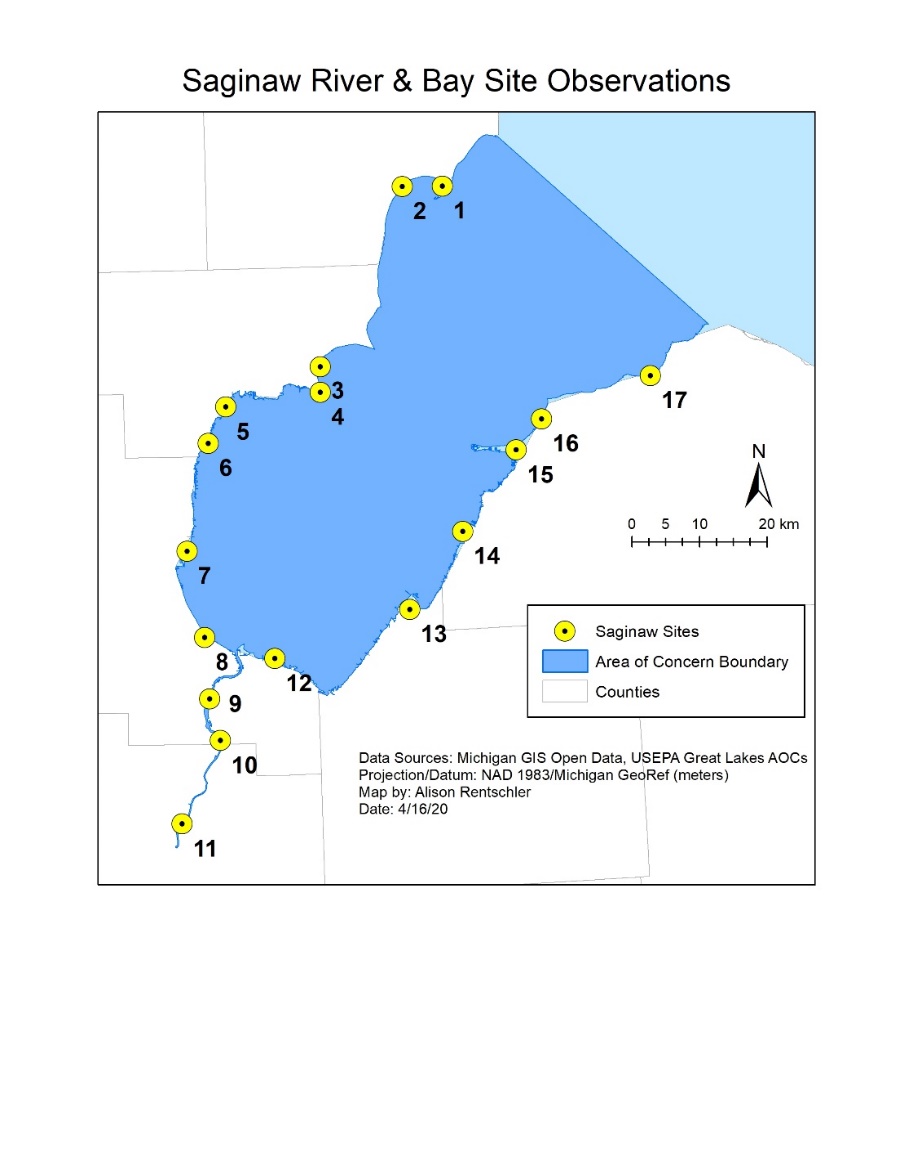

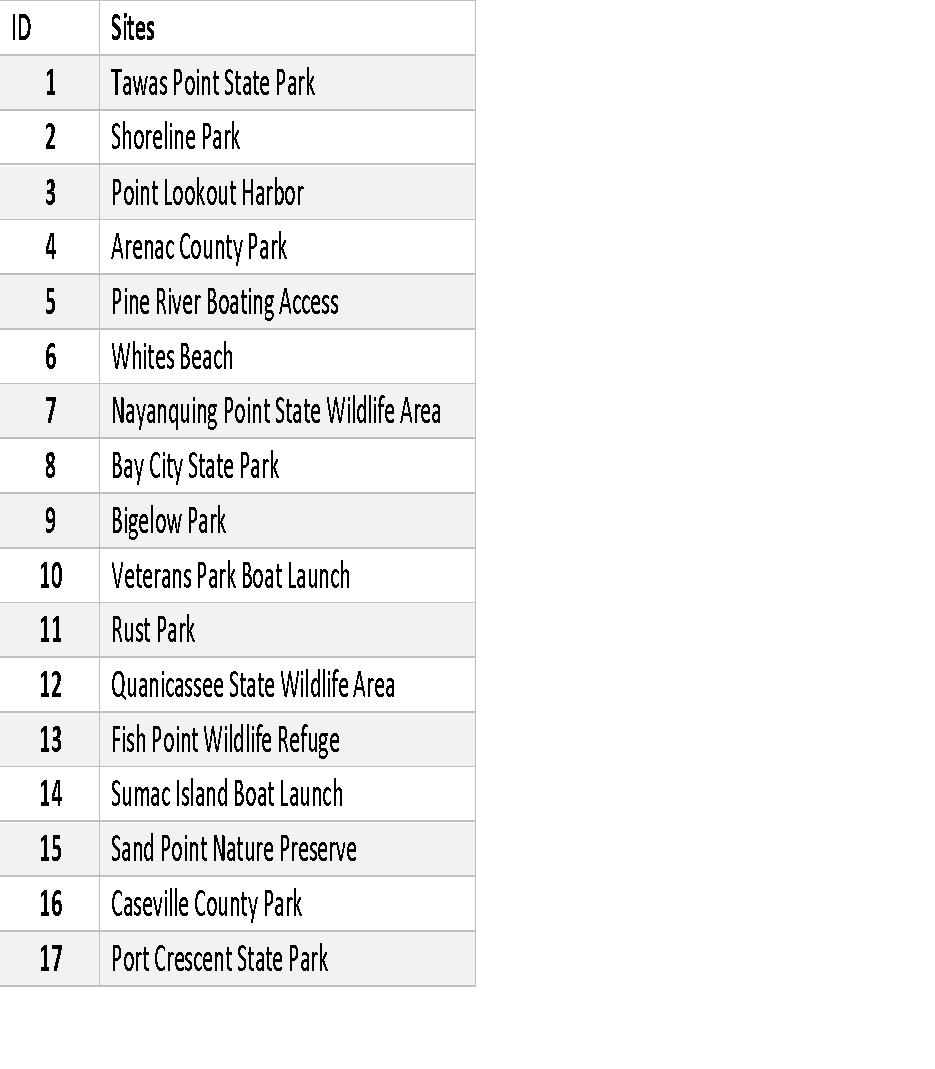


Figure S3. Rouge River Site Observations and AOC Extent Map


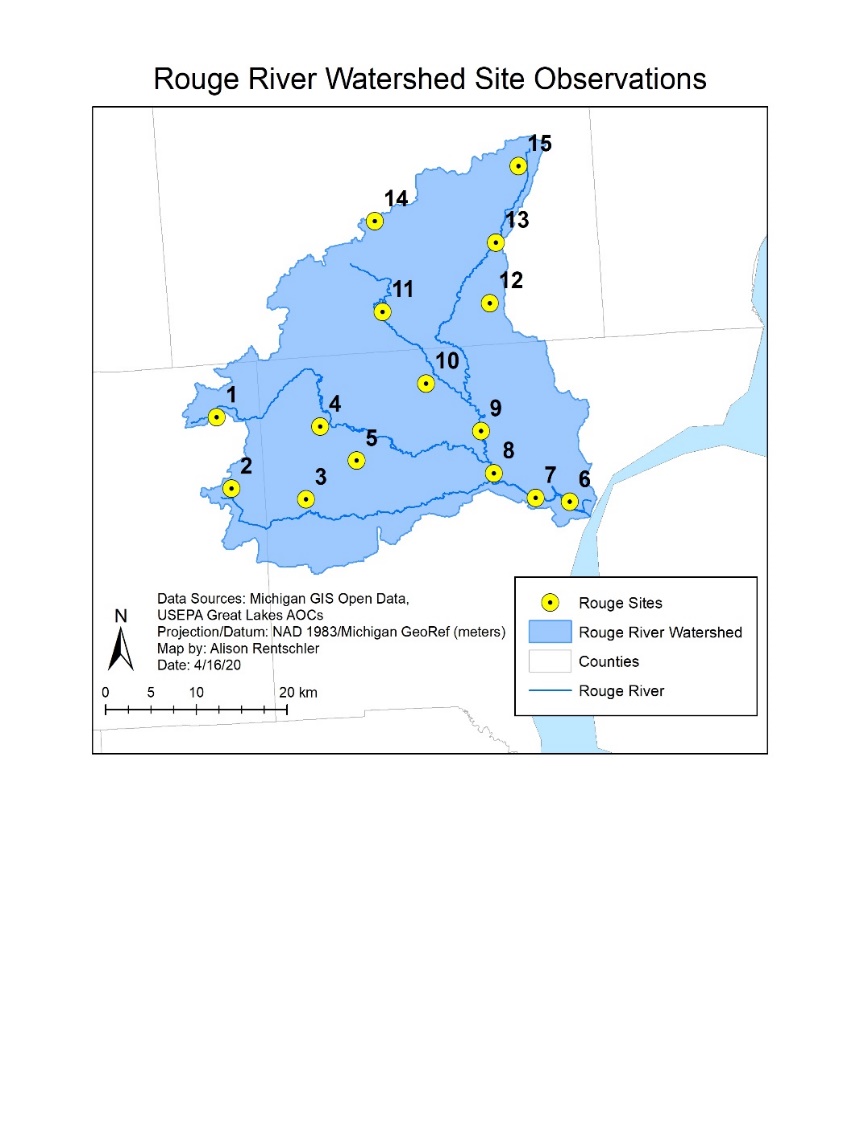

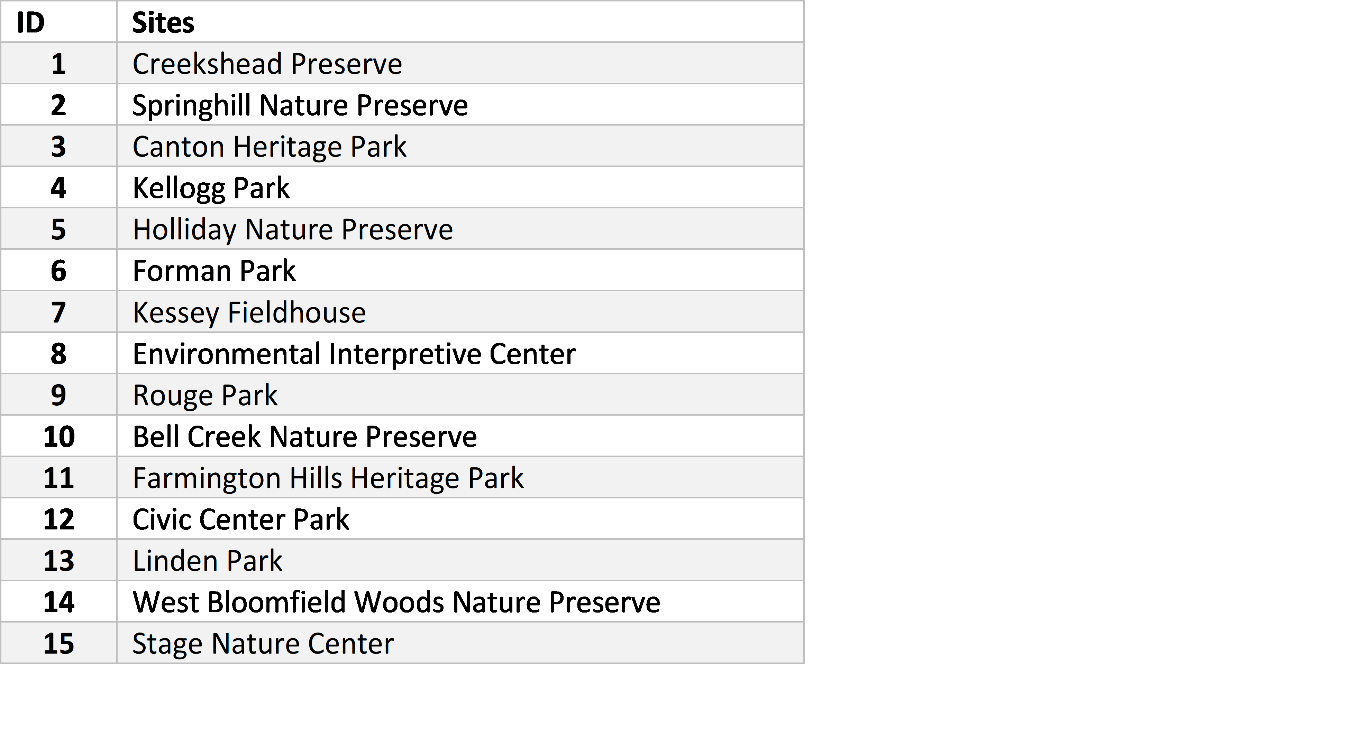


Table S1. Participatory Observation Locations

|  | **Sites** | **Meetings** | **Events** |
| --- | --- | --- | --- |
| **Kalamazoo River** | - Mayors Riverfront Park - Arcadia Brewing Company - Verburg Park - Markin Glen Park - Kalamazoo Nature Center - Gilkey Elementary School Trails - Plainwell City Hall - Former Otsego Township Dam - Hanson Park - Allegan Dam - Mount Baldhead Park - Saugatuck Dunes State Park - Portage Creek | - Kalamazoo River Watershed Council (KRWC) Board Meeting (6/17/19) - KRWC Board Meeting (8/19/19) | - KRWC: Kanoe the Kazoo - Fort Custer Recreation Area (6/18/19) |
| **Saginaw River & Bay** | - Tawas Point State Park - Shoreline Park - Point Lookout Harbor - Arenac County Park - Pine River Boating Access - Whites Beach - Nayanquing Point State Wildlife Area - Bay City State Park - Bigelow Park - Veterans Park Boat Launch - Rust Park - Quanicassee State Wildlife Area - Fish Point Wildlife Refuge - Sumac Island Boat Launch - Sand Point Nature Preserve - Caseville County Park - Port Crescent State Park | - Partnership for the Saginaw Bay Watershed (PSBW) Board Meeting (7/9/19) - PSBW Board Meeting (8/6/19) | - Little Forks Conservancy: Invasive Species Hike – Averill Preserve (6/6/19) - Friends of the Shiawassee River: Shiawassee River Cleanup (7/27/19) |
| **Rouge River** | - Creekshead Preserve - Springhill Nature Preserve - Canton Heritage Park - Kellogg Park - Holliday Nature Preserve - Forman Park - Kessey Fieldhouse - Environmental Interpretive Center - Rouge Park - Bell Creek Nature Preserve - Farmington Hills Heritage Park - Civic Center Park - Linden Park - West Bloomfield Woods Nature Preserve - Stage Nature Center | - Friends of the Rouge (FOTR) Board Meeting (6/12/19) - FOTR Water Trail Committee Meeting (6/20/19) - RRAC Board Meeting (8/27/19) | - FOTR: Rain Garden Install – Plymouth (6/15/19) - FOTR: Rain Garden Install – FOTR Headquarters (6/24/19) - FOTR: Trash2Art River Cleanup (7/2/19) - FOTR: Eliza Howell Park Discovery Day (7/13/19) - FOTR: Rouge Cruise (8/6/19) |

Figure S4. Site Observation Template


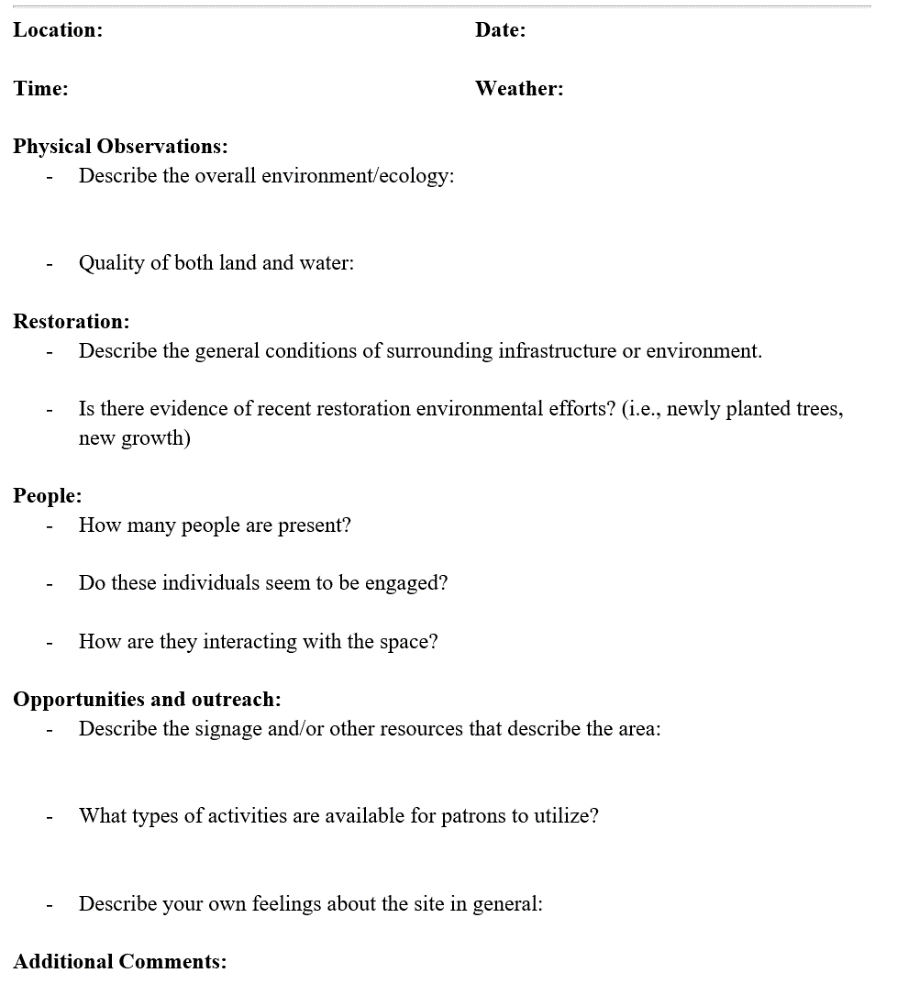


Figure S5. Meeting Observation Template


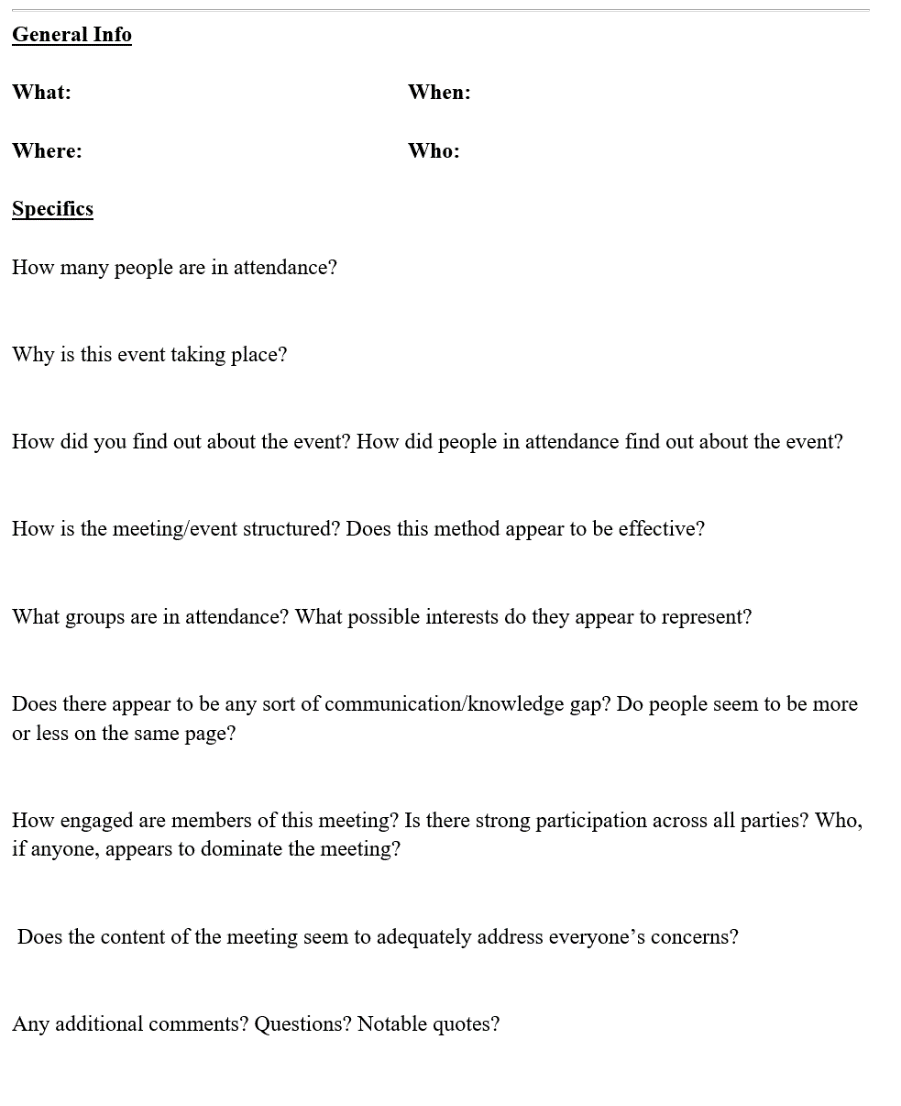


Figure S6. Event Observation Template


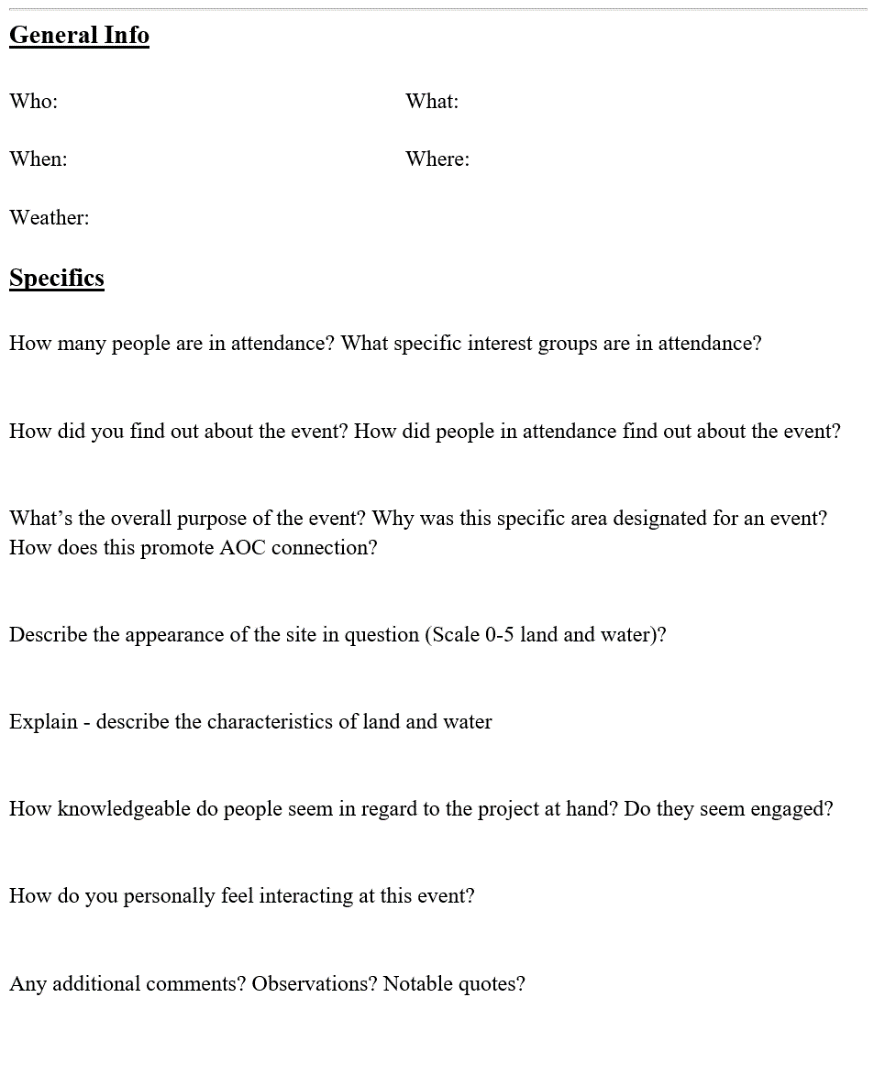


Figure S7. PAC Interview Template


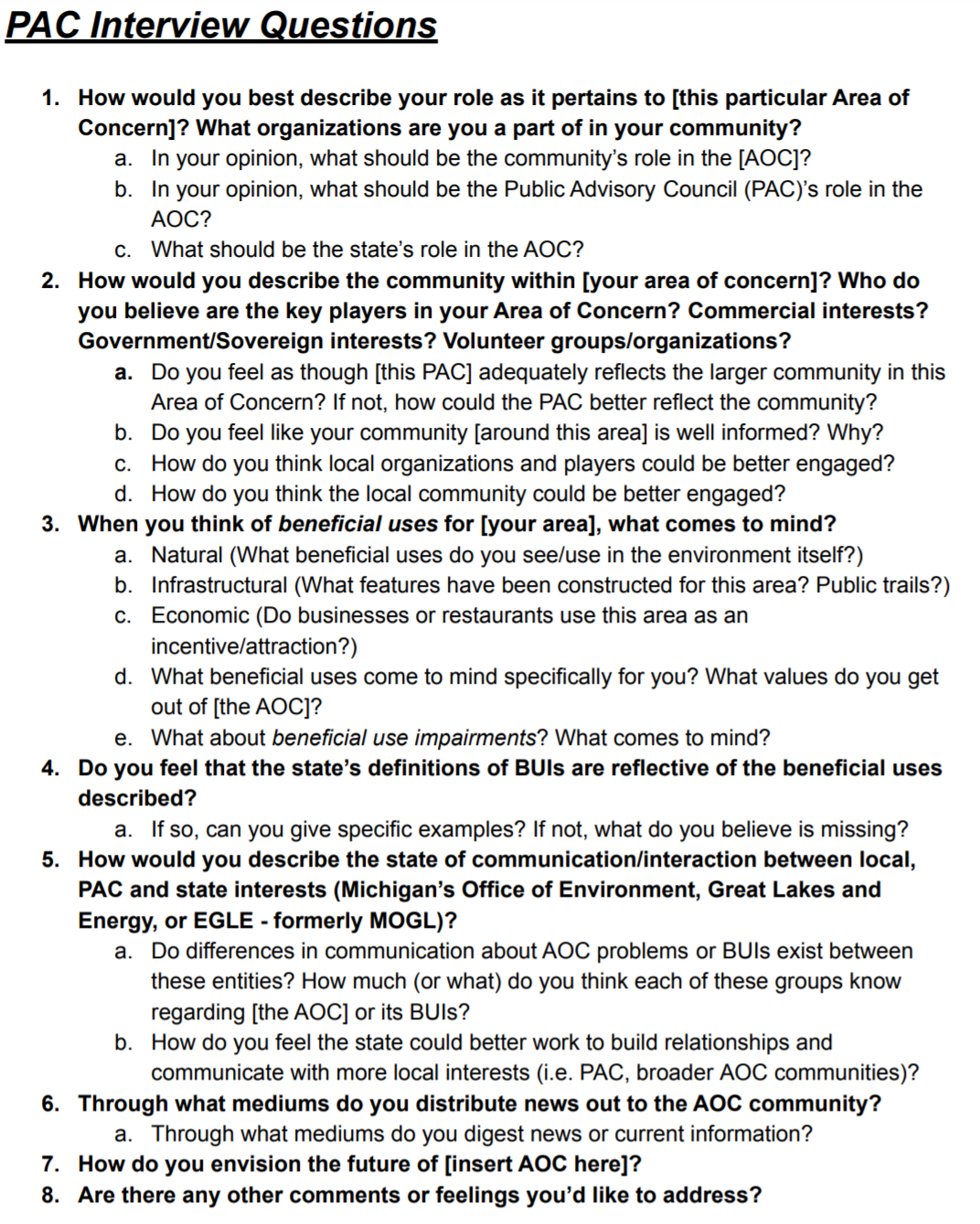


Figure S8. EGLE Interview Template


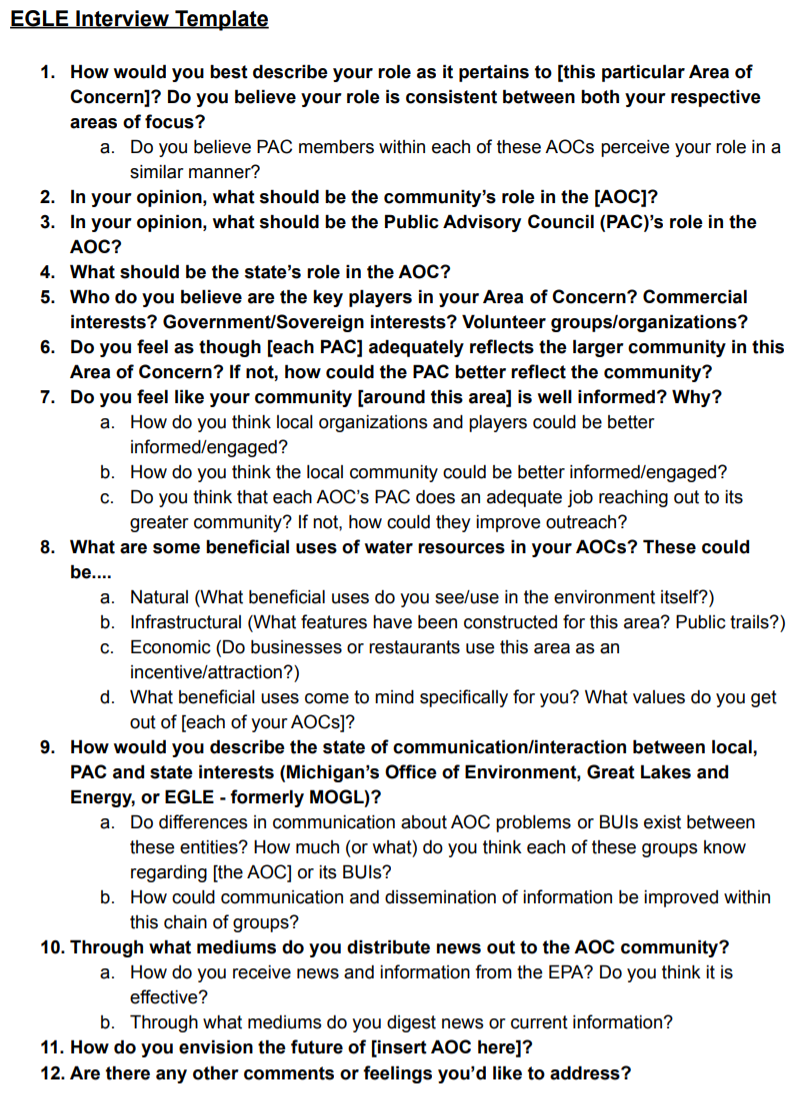


Figure S9. Focus Group Template


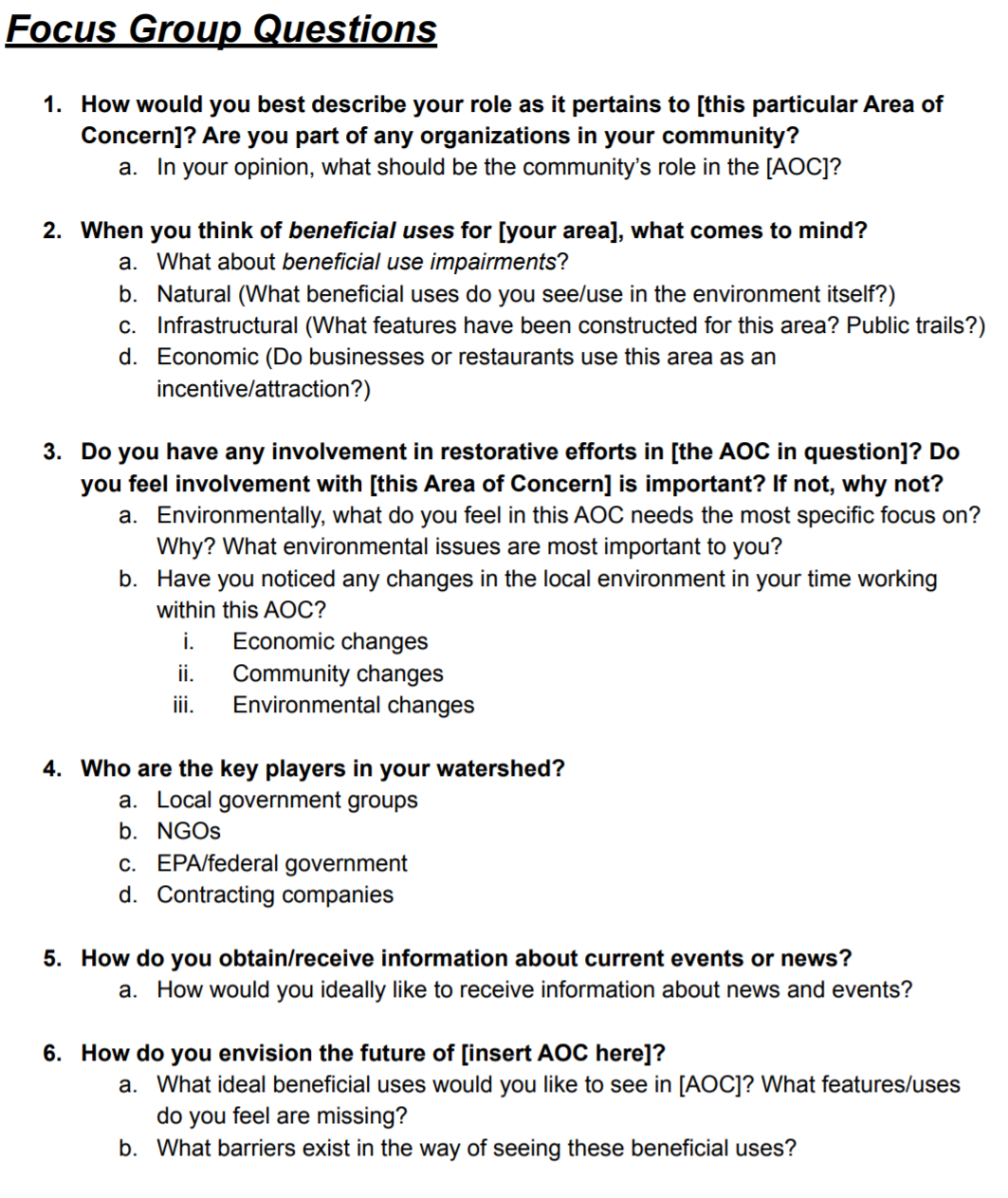


Table S2. Inductive Codebook

**
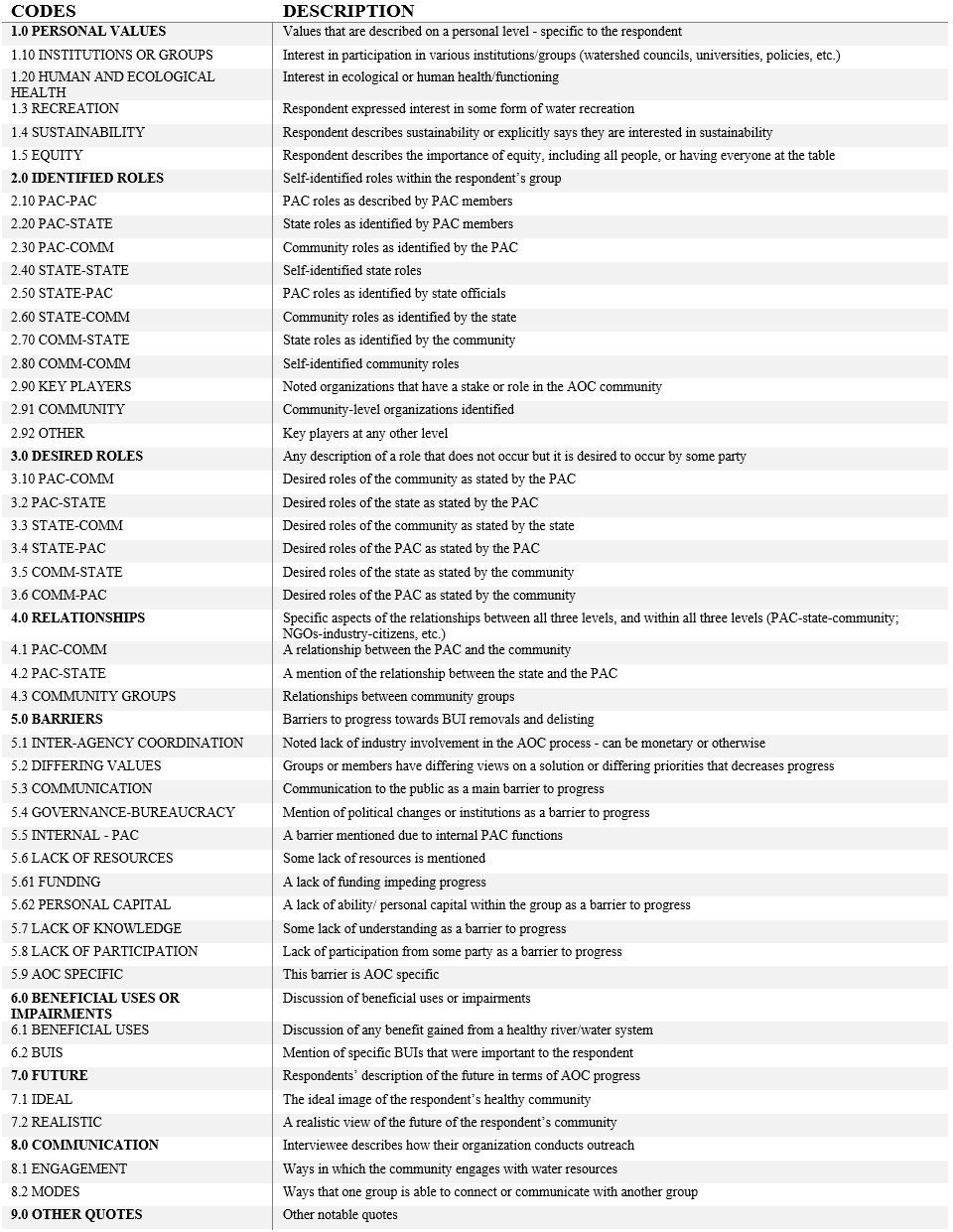
**

Figure S10. Neighborhood Model (Williams et al., 2018)


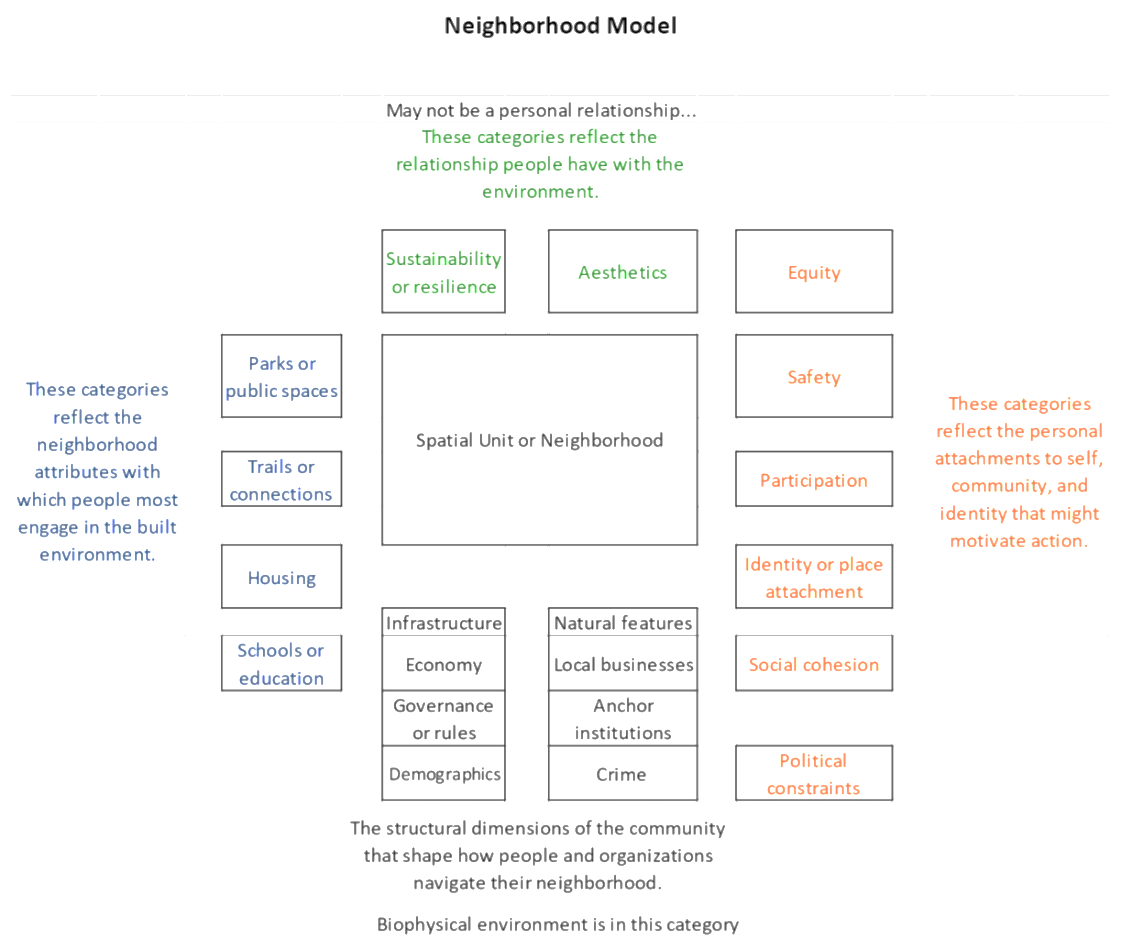

Supplement: SI [file NIHMS1839616-supplement-SI.docx]
